# Dose-Dependent Responses of *Escherichia coli* and *Acinetobacter* sp. to Micron-Sized Polystyrene Microplastics

**DOI:** 10.4014/jmb.2410.10023

**Published:** 2025-02-24

**Authors:** So Yoon Kim, Shinyoung Woo, Seung-Woo Lee, Eui-Man Jung, Eun-Hee Lee

**Affiliations:** 1 Department of Microbiology, Pusan National University, Busan 46241, Republic of Korea; 2 Institute for Future Earth, Pusan National University, Busan 46241, Republic of Korea; 3 Department of Fine Chemistry, Seoul National University of Science and Technology, Seoul 08826, Republic of Korea; 4 Center for Functional Biomaterials, Seoul National University of Science and Technology, Seoul 08826, Republic of Korea; 5 Department of Molecular Biology, Pusan National University, Busan 46241, Republic of Korea

**Keywords:** Bacterial growth, viability, oxidative stress, microbeads

## Abstract

Microplastics are ubiquitous environmental contaminants that can cause significant ecological damage because of their resistance to biodegradation. We evaluated the toxic effects of 1,040 nm polystyrene (PS) microplastics in two representative bacteria, *Escherichia coli* and *Acinetobacter* sp. In particular, we examined the effects of these PS microplastics on bacterial growth and viability, parameters related to oxidative stress (reactive oxygen species [ROS], lactate dehydrogenase [LDH], and malondialdehyde [MDA]), membrane integrity, and biofilm formation. An increasing concentration of PS microplastics decreased cell growth and viability in both species. These PS microplastics also decreased cell membrane integrity and increased biofilm formation in both species. Although both species exhibited adverse overall effects from PS microplastics, they had significant differences in specific indicators of oxidative stress. Correlation analysis demonstrated different correlations among measured experimental parameters (cell viability, ROS, LDH, MDA, and biofilm formation) in these two species. These results suggest that 1,040 nm PS microplastics decreased cell growth and viability by different mechanisms in *E. coli* and *Acinetobacter* sp.

## Introduction

Plastics are artificial synthetic or semi-synthetic polymers that are typically inexpensive, lightweight, durable, and easy to produce [1]. For these reasons, industries commonly use plastics to manufacture beverage bottles, food packaging, medical devices, appliances, and many other products [2-4]. However, the increasing use of plastics has led to an increasing amount of plastic waste, and plastic pollution is now a global environmental problem.

The improper disposal of plastic waste allows this waste to enter the oceans due to transport by wind, precipitation, and runoff from rivers and streams [5]. Over time, most plastics fragment into smaller particles, including microplastics (<5 mm), through exposure to ultraviolet radiation, corrosion, and physico-chemical processes [6]. These microplastics are highly resistant to degradation, undergoing slow breakdown in the environment [7]. In fact, microplastics can persist for a long period in the environment, and they have adverse impacts on human health and ecosystems [8]. Moreover, the degradation of microplastics leads to the formation of nanoplastics (<1 μm), which are also harmful [9].

Nano- and microplastics are now pervasive in many environments, and numerous studies have reported their presence in aquatic, terrestrial, and atmospheric ecosystems [10-12]. Microbial cells can adhere to microplastics, while they can uptake nanoplastics inside cells [13]. Most studies of microbial interactions with microplastics have analyzed microbial communities [14-16].

Previous studies of bacteria have reported that nanoplastics are more likely than microplastics to be toxic and impair cellular functions [2, 17]. Specifically, smaller polystyrene (PS) microplastics have more inhibitory effects on bacterial growth, and they also induce oxidative stress and disrupt cell membrane integrity [18, 19]. Ning *et al*.(2021) found that 30 nm PS nanoplastics inhibited the growth of *Escherichia coli* [20]. Sun *et al*. (2018) studied the effect of different PS particles (320 mg/l) on the *Halomonas alkaliphila* and found that 50 nm particles were more harmful than 1 μm particles. In addition, *E. coli* cells exposed to PS particles (80 mg/l) had lower viability when these particles were 0.1 μm rather than 0.55 and 5 μm.

Our previous study investigated the effects of PS particles with varying diameters (60, 220, 430, 700, 1,040, 1,700, and 2,260 nm) on bacterial growth and viability [13]. The results revealed size-dependent interactions between PS particles and bacterial cells. Specifically, exposure to smaller PS particles (60 nm) significantly increased reactive oxygen species (ROS) levels in *E. coli* without substantially impacting bacterial growth. In contrast, larger particles (>1,700 nm) exhibited weaker inhibitory effects on growth and ROS production. Notably, the 1,040 nm PS microplastics demonstrated the greatest inhibition of *E. coli* growth, despite causing only a modest increase in ROS levels. This differential response is somewhat inconsistent with other reports, indicating the need for further research to elucidate this effect in more detail.

This study aimed to evaluate the effects of 1,040 nm PS microplastics at different concentrations (0–100 mg/l) on *E. coli* and *Acinetobacter* sp. Specifically, we examined the contributions of oxidative stress–including lipid peroxidation and membrane damage–and biofilm formation to the observed bacterial responses. By assessing key markers such as ROS, lactate dehydrogenase (LDH), malondialdehyde (MDA), and membrane integrity, the study provides foundational insights into bacterial interactions with 1,040 nm PS microplastics. Additionally, we conducted pairwise correlation analyses of these parameters within each species and compared the resulting correlation patterns to further elucidate the interactions involved.

## Materials and Methods

### Cell Growth

The bacterial strains used in this study were *E. coli* and *Acinetobacter* sp. These strains were selected due to their frequent detection on plastic waste surfaces in the environment and their potential for colonization and interaction with plastics [21, 22]. The bacterial cells were cultivated in a nutrient broth (MBcell, KisanBio Co., Republic of Korea) at 37°C for *E. coli* (KCTC 2791) and 30°C for *Acinetobacter* sp. (OQ402585) with an agitation at 150 rpm for 16 h [13]. Then, a 500 μl sample (cell concentration: 8.5 ± 1.8 to 8.1 ± 0.2 × 10^8^ colony forming units [CFU]/ml) was inoculated into a 125-ml Erlenmeyer flask that contained a nutrient broth of 49.4 ml. Different aliquots of spherical PS microplastics were added to achieve final concentrations of 0 (negative control), 10, 25, 50, 75, and 100 mg/l. The PS microplastics, with a diameter of 1,040 nm, were purchased from Spherotech Inc.(Catalog no. PP-10-10, 5% (w/v), non-functionalized surface, USA). Then, each 50 ml sample was cultivated at 37°C for 9 h (*E. coli*) and at 30°C for 11 h (*Acinetobacter* sp.) with shaking at 150 rpm. The optical density of each culture broth was acquired at 600 nm (OD_600_) at intervals of 1 to 3 h using a spectrophotometer (UV-1800, Shimadzu Co., Japan), and expressed relative to the OD_600_ of the negative control (OD_600, NC_) that had no microplastics:

$$\text{Relative }OD_{600\;nm}(\%)=\frac{OD_{600,\;Sample}}{OD_{600,\text{ NC}}}\times 100$$ (1)

The experiments were carried out in triplicate unless otherwise mentioned.

### Cell Viability

The same basic procedures were used to prepare cells for measurements of cell viability, ROS, LDH, and MDA unless otherwise mentioned. The culture broths of *E. coli* and *Acinetobacter* sp. were collected at 13,200 g for 10 min, and then rinsed off twice with PBS (0.1 M, pH 7.4). The collected cells were resuspended in fresh PBS buffer to a concentration of 8.5 ± 1.8 × 10^8^ CFU/ml (*E. coli*) and 8.1 ± 0.2 × 10^8^ CFU/ml (*Acinetobacter* sp.). An aliquot (49.9 ml) of each culture was transferred into a 125-ml flask, and 100 μl of PS microplastics were added to achieve final concentrations of 0 to 100 mg/l, followed by incubation at 37°C (*E. coli*) and 30°C (*Acinetobacter* sp.). After 24 h, samples were subjected to serial dilution with 0.9% NaCl. Aliquots of each diluted culture broth were inoculated onto nutrient agar plates, prepared by adding agar (Duchefa Biochemie, The Netherlands) to nutrient broth, and cultivated at 37°C for *E. coli* and 30°C for *Acinetobacter* sp. for 16 h. The colonies on these plates were counted, and cell viability was expressed as CFU/ml (CFU/ml, _Sample_) relative to the negative control (CFU/ml, _NC_) that had no microplastics:

$$\text{Cell viability(\%)=}\frac{CFU/ml{,}_{Sample}}{CFU/ml{,}_{NC}}\times 100$$ (2)

### ROS Assay

The levels of ROS were evaluated after exposure of cells to different concentrations of PS microplastics (0–100 mg/l) for 24 h. In this assay, a 960 μl aliquot of each culture was transferred into a 2 ml tube, and a DCF-DA solution (Sigma-Aldrich, USA) was added to achieve a desired concentration of 80 μM [13]. The tubes were then incubated in the dark at ambient temperature with agitation at 200 rpm for 30 min. The positive control used a 50 μl aliquot of a hydrogen peroxide solution (0.05% H_2_O_2_, Sigma-Aldrich). Then, 300 μL was collected from each tube, transferred into a 96-well plate, and fluorescence was acquired at an emission band of 535 nm (F_535_) using a spectrofluorometer (SpectraMax M2, Molecular Devices, USA) with an excitation at 488 nm. The ROS level was expressed relative to a negative control (F_535, NC_) that had no microplastics:

$$\text{Relative }ROS\text{(\%)=}\frac{F_{535,\;Sample}}{F_{535,\text{ NC}}}\times 100$$ (3)

### LDH Assay

The effect of different concentrations of PS microplastics on LDH release was measured using the CyQUANT^TM^ LDH Cytotoxicity assay kit (Thermo Fisher Scientific, USA) according to the manufacturer’s instruction. After incubation for 24 h, 50 μl of the supernatant was added to a 96-well plate. For maximum LDH release, 10^8^ CFU/ml cells were lysed in an ice bath using a tip sonicator (VCX 750, Sonics & Materials, Inc., USA) at a duty cycle of 16.7% (10 s sonication and 50 s rest) for 20 min. The sonicated sample was added to a 96 well-plate, followed by addition of 50 μl of the substrate mixture and incubation without agitation at ambient temperature in the dark for 30 min. Then, 50 μl of the stop solution was added, absorbance was acquired with a spectrophotometer at 490 nm and 680 nm (background), and the difference in absorbance at these two wavelengths was determined. The LDH release was calculated using the LDH activity of the PS-treated sample (LDH_T_), spontaneous LDH activity (LDH_S_), and maximum LDH activity (LDH_M_):

$$LDH(\%)=\frac{LDH_{T}-LDH_{S}}{LDH_{M}-LDH_{S}}\times 100$$ (4)

The LDH release of each sample (LDH_Sample_) was then expressed relative to the negative control (LDH_NC_) that had no microplastics:

$$\text{Relative }LDH(\%)=\frac{LDH_{Sample}}{LDH_{NC}}\times 100$$ (5)

### MDA Assay

Bacteria were cultivated in the same manner as described in the Cell Viability section. After 24 h of incubation, lipid peroxidation was determined using an MDA assay kit (Sigma-Aldrich) in accordance with the manufacturer’s instructions. Two hundred fifty μl of 1% *tert*-butyl hydroperoxide solution (t-BHP, Sigma-Aldrich) was added to a 4.75 ml aliquot of bacterial culture as a positive control. A 5 ml aliquot of each sample was sonicated as described in the LDH Assay section, and the lysed cells were collected at 4,355 *g* for 10 min. Solutions with different concentrations of MDA (0–10 nM) were used to establish a calibration curve. Two hundred μl of the supernatant was mixed with 600 μl of 0.8% trichloroacetic acid (Sigma-Aldrich) in 30% glacial acetic acid (Duksan Co., Republic of Korea), and incubated in a heat block at 95°C for 1 h. The reaction mixture was then cooled on ice for 10 min, a 200 μl aliquot of the supernatant was transferred into a 96-well plate, absorbance was acquired at 532 nm, and the MDA content was determined from the calibration curve.

The MDA content of each sample (MDA_Sample_) was expressed relative to the negative control (MDA_NC_) that had no microplastics:

$$\text{Relative }MDA\;content(\%)=\frac{MDA_{Sample}}{MDA_{NC}}\times 100$$ (6)

Each experimental treatment had 5 replicates.

### Biofilm Formation

First, a 500 μl sample from the pre-culture (as described in the Cell Growth section) was added to a 125 ml flask that contained a fresh nutrient broth. Then, PS microplastics were added to achieve final concentrations of 0 to 100 mg/l. A 25 ml aliquot of each reactant solution was incubated at 37°C for *E. coli* and 30ºC for *Acinetobacter* sp. with shaking at 150 rpm for 24 h. Then, a 1 ml aliquot of each culture was centrifuged at 3381 g for 3 min, and the collected cells were stained for 15 min with 0.1% crystal violet (Samchun Chemical Co. Ltd., Republic of Korea). Then, the unbound crystal violet was rinsed off twice with deionized water, and the sample was air-dried for 24 h. The bound crystal violet was dissolved using 200 μl of 30% acetic acid (Duksan Co.) for 15 min, a 150 μl was added to a 96-well plate, and absorbance was acquired at 550 nm (OD_550_) using a plate reader. The biofilm formation was calculated relative to a negative control (OD_550, NC_) that had no microplastics:

$$\text{Relative biofilm formation(\%)=}\frac{OD_{550,\;Sample}}{OD_{550,\;NC}}\times 100$$ (7)

### Cell Membrane Integrity Assay

A 500 μl aliquot of pre-cultured cells (as described in the Cell Growth section) was inoculated into a nutrient broth, and PS microplastics were added to be a desired concentration of 50 mg/l. Cells without microplastics were used as the negative control. Then, 10 ml of cells were cultivated at 37°C for *E. coli* and 30°C for *Acinetobacter* sp. with shaking at 150 rpm. After 24 h, a 1 ml sample was collected at 17,177 g for 3 min. The collected cells were rinsed off twice with PBS buffer, and then suspended in fresh PBS buffer. Each sample was stained with 500 μl of propidium iodide (50 μg/ml PI, Sigma-Aldrich) for 15 min at ambient temperature in the dark, and then 20 μl was added onto a slide glass and topped with a cover glass. The samples were air-dried and observed using a fluorescence microscope (Axio Observer 3, Zeiss, Germany) with a rhodamine filter (excitation: 551–556 nm, emission: 582–595 nm).

### Measurements of Hydrodynamic Diameter and Zeta Potential

Samples that had different mixtures of PS microplastics and cells were prepared (Table 1). Each sample was then transferred into a flow cell (Otsuka Electronics, Japan), and the surface charge and hydrodynamic diameter were determined using laser Doppler velocimetry using a zeta-potential and particle size analyzer (ELS-Z2, Otsuka Electronics).

### Statistical Analysis

Student’s *t*-test was employed to assess the significance of differences between the means of two samples (*e.g.*, bacteria alone [negative control] vs. bacteria with PS microplastics) using Sigmaplot (Systat Software, Inc., USA). In all cases, a *p*-value below 0.05 was deemed significant and is denoted with an asterisk (*).

Data regarding cell viability; levels of ROS, LDH, and MDA; and biofilm formation were compiled in Microsoft Excel (Microsoft Co., USA), with each dataset corresponding to measurements of a specific parameter. These raw data were subjected to a preprocessing procedure to ensure consistency and reliability for subsequent analysis; this consisted of removal of non-numeric entries, and matching of data points according to the concentration of PS microplastics.

Pearson correlation coefficients were calculated to assess the linear relationships among the different parameters and provide insights into their possible causal relationships. The correlation coefficient was computed using Python programming (Python Software Foundation, USA); a value of 1 represented a perfect positive correlation, a value of −1 represented a perfect negative correlation, and a value of 0 indicated no correlation. The *p*-value of each correlation coefficient was also calculated, and a *p*-value below 0.05 indicated a significant correlation.

For each dataset, a correlation heatmap was generated to display the correlation coefficients. This heatmap was generated using the Python programming language, with libraries such as Pandas, Matplotlib, and Seaborn.

## Results and Discussion

### Effect of Different Concentrations of PS Microplastics on Cell Growth and Viability

**Fig. 1:**
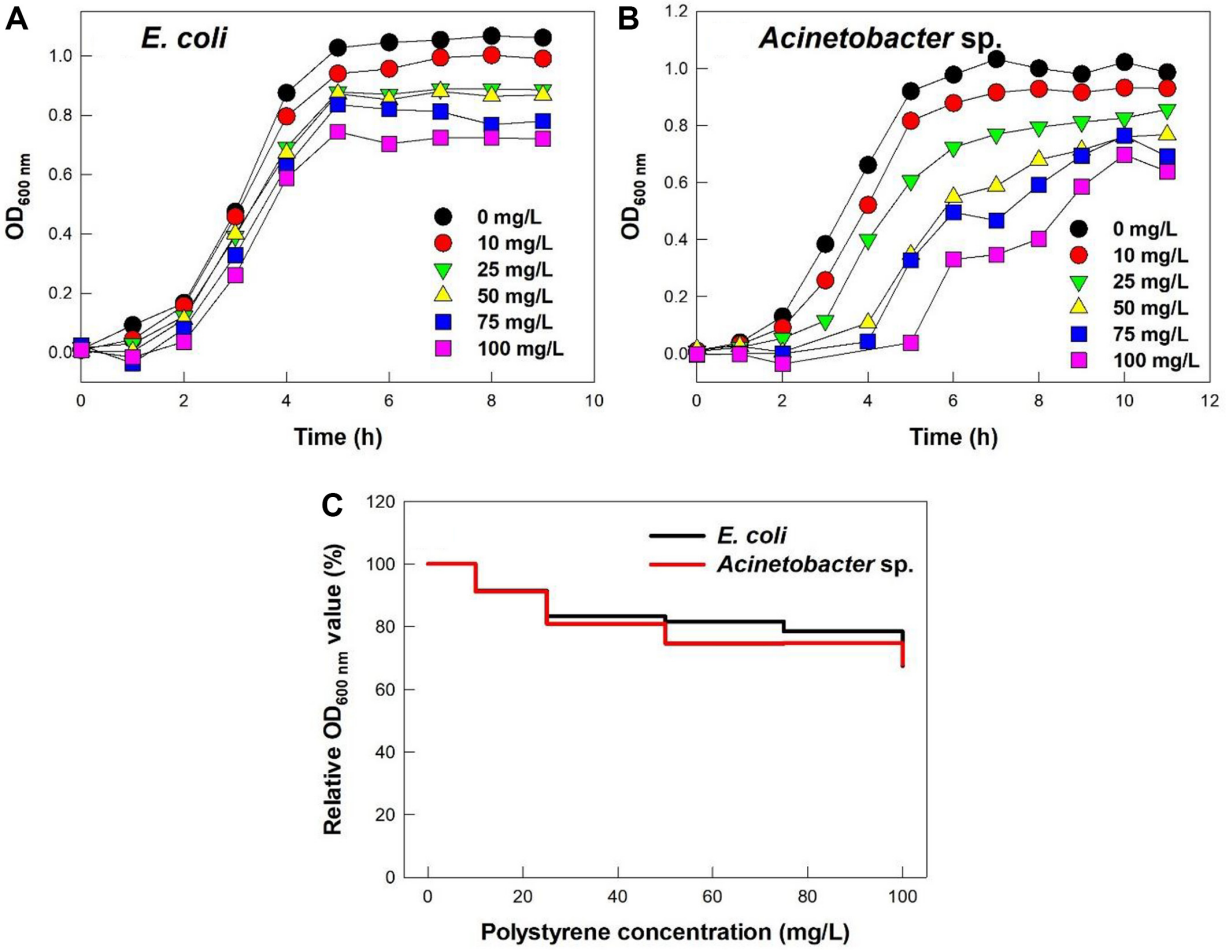
Effect of PS microplastic concentration on (A, B) growth and (C) growth relative to controls without PS microplastics at 6 h and 10 h in *E. coli* and *Acinetobacter* sp.

The PS microplastics inhibited the growth of *E. coli* and *Acinetobacter* sp. in a dose-dependent manner, but the two species differed in the pattern of inhibition (Fig. 1A and 1B). For all tested PS microplastic concentrations, *E. coli* cells began exponential growth after a 2 h-lag phase and reached a stationary phase at about 5 h (Fig. 1A). After 6 h, cell growth (OD_600_) relative to the control without PS microplastics decreased in a dose-dependent manner from 91.5% at 10 mg/l to 67.4% at 100 mg/l (Fig. 1C).

On the other hand, the lag phase of *Acinetobacter* sp. cells increased with increasing concentrations of PS microplastics (Fig. 1B). At concentrations of 0 and 10 mg/l, the lag phase was about 2 h; at a concentration of 25 mg/l, the lag phase was about 3 h; at concentrations of 50 and 75 mg/l, the lag phase was about 4 h, and at a concentration of 100 mg/l, the lag phase was about 5 h. In addition, *Acinetobacter* sp. cells exhibited a slightly diauxic response at concentrations of 75 and 100 mg/l, with intermediate lag phases of 1 to 2 h. After 10 h, the cell growth (OD_600_) relative to the control without PS microplastics decreased in a dose dependent manner from 91.2%at 10 mg/l to 68.0% at 100 mg/l (Fig. 1C) very similar to *E. coli*.

**Fig. 2:**
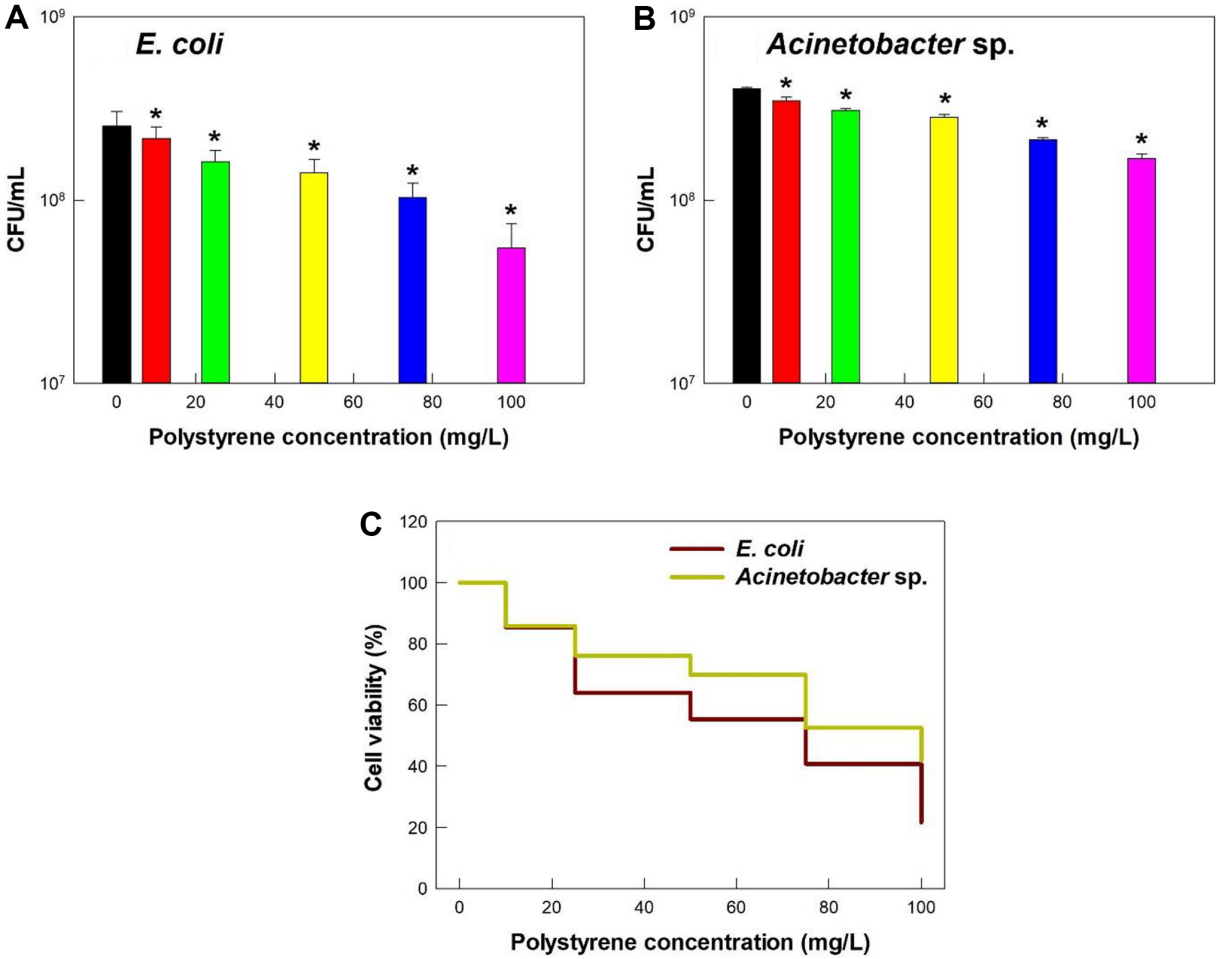
Effect of PS microplastic concentration on (A, B) viability of *E. coli* and *Acinetobacter* sp. and (C) viability relative to controls without PS microplastics. Here and below: an asterisk (*) indicates a significant difference between a sample and the negative control (*p* < 0.05).

Incubation of cells with PS microplastics also decreased the viability of *E. coli* and *Acinetobacter* sp. in a dose-dependent manner (Fig. 2A and 2B). However, for a concentration of 100 mg/l, the viability was 21.6% in *E. coli* and 41.8% in *Acinetobacter* sp. (Fig. 2C).

Sun *et al*. (2020) also reported decreased viability of *E. coli* cells when exposed to 1 μm PS microplastics [23]. In particular, they found that exposure to 1 μm PS microplastics for 24 h slightly decreased viability at a concentration of 100 mg/l, but had a smaller effect at concentrations of 1 to 30 mg/l. Ustabasi and Baysal (2020) found a slight decrease in the growth of *E. coli* upon exposure to polyethylene (PE) microplastics, but doses in the range of 10 to 500 mg/l had similar effects [24]. Similar to our study, Yi *et al*. (2021) observed a dose-dependent decrease in cell viability of *E. coli* upon exposure to PS microplastics that had diameters of 0.1, 0.55, and 5 μm [25].

In the case of gram-positive bacteria, their cellular growth and viability may differ from those of gram-negative bacteria. For instance, Yi *et al*. (2021) reported the enhanced growth of the gram-positive *Bacillus cereus* upon the addition of PS microplastics, which might be attributed to differences in cell wall composition and the distinct interactions between the bacteria and PS particles [25]. Conversely, Cheng *et al*. (2023) documented that *B. subtilis* experienced inhibition in the presence of PS microplastics, similar to the behavior observed in gram-negative bacteria in our study [26]. Taken together with our findings, these results suggest that the inhibitory effects of micron-sized PS microplastics may vary depending on the composition of bacterial cell wall structures. Furthermore, they indicate that the adverse effects of microplastics depend on the bacterial species, polymer type, and polymer size.

### Effect of Different Concentrations of PS Microplastics on the Levels of ROS, LDH, and MDA

**Fig. 3:**
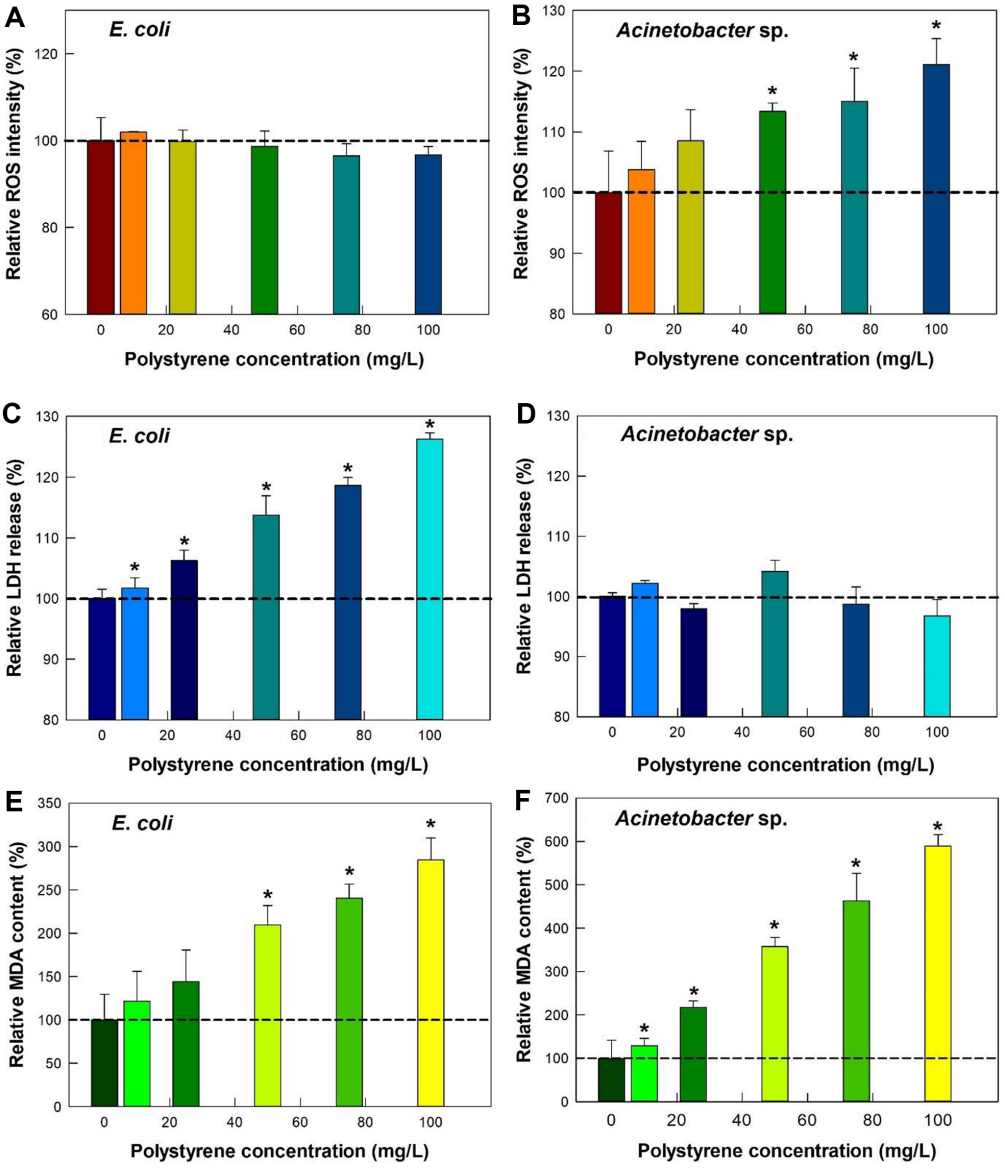
Effect PS microplastic concentration on (A, B) ROS production, (C, D) LDH release, and (E, F) MDA level in *E. coli* and *Acinetobacter* sp.

We then examined the effects of 1,040 nm PS microplastics at various concentrations on the ROS level, LDH release, and MDA content of *E. coli* and *Acinetobacter* sp. (Fig. 3). Our results showed distinct responses between the two species. In *E. coli*, PS microplastics increased LDH release in a dose-dependent manner (Fig. 3C) but had no significant effect on ROS levels up to 100 mg/ml (Fig. 3A). Conversely, *Acinetobacter* sp. exhibited a dose-dependent increase in ROS levels (Fig. 3B) without significant LDH release up to 100 mg/ml (Fig. 3D). The MDA content, indicative of lipid peroxidation, increased in both species in a dose-dependent manner, but this increase was approximately twice as high in *Acinetobacter* sp. as in *E. coli* (Fig. 3E and 3F).

Although PS is a chemically inert plastic, its interaction with bacterial cells likely triggers oxidative stress responses through physical mechanisms. These include membrane disruption, as supported by increased LDH release (Fig. 3C and 3D), and lipid peroxidation, as indicated by elevated MDA levels (Fig. 3E and 3F).

**Fig. 4:**
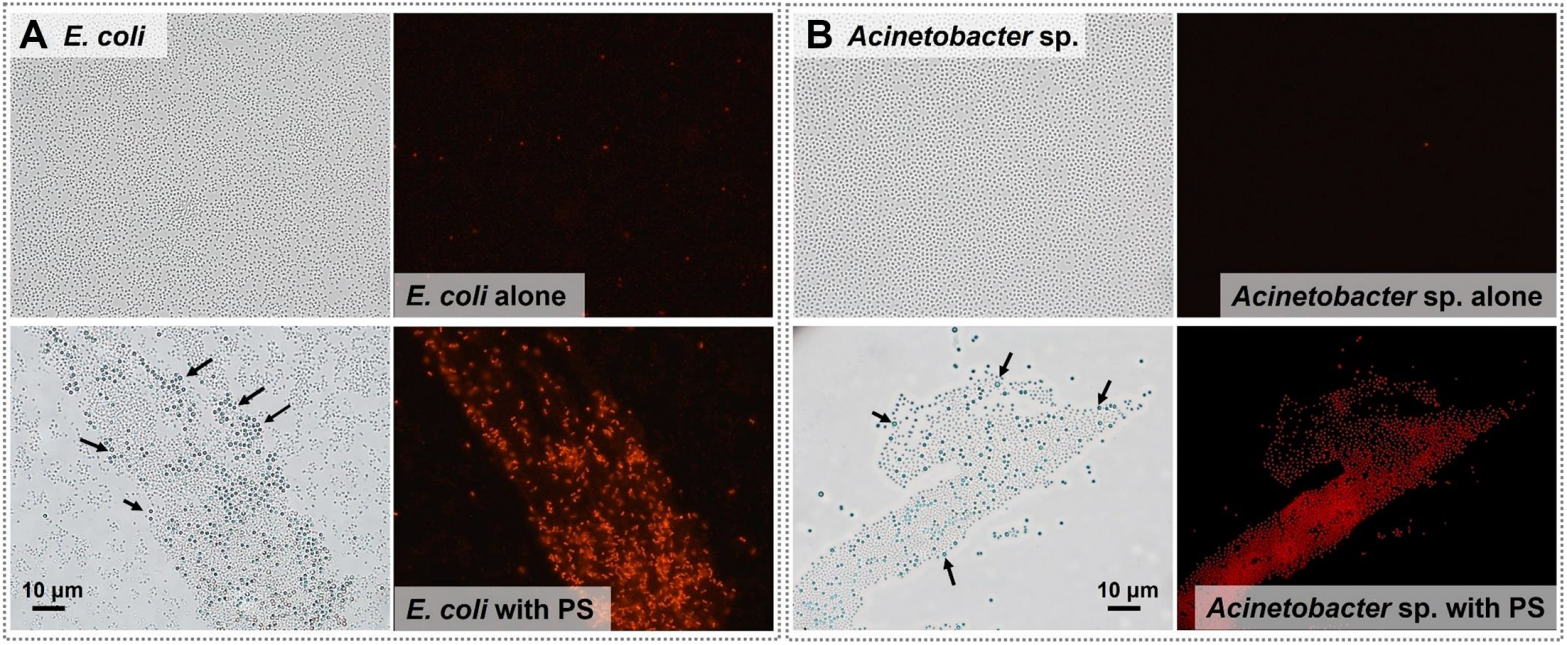
Effect of 50 mg/l PS microplastics on membrane integrity of (A) *E. coli* and (B) *Acinetobacter* sp. based on fluorescence microscopy. Arrows indicate PS microplastics.

We also used microscopy to examine the effect of PS microplastics on cell membrane integrity (Fig. 4). *E. coli* and *Acinetobacter* sp. cells clearly exhibited red fluorescence upon exposure to 50 mg/l of PS microplastics compared to the negative control (cells alone). This enhanced fluorescence was present in bacterial cells that surrounded the PS microplastics (spheres and arrows in Fig. 4) and indicated damage of cell membrane integrity.

Oxidative stress is a common consequence of membrane disruption, as it disrupts membrane-associated enzymes and promotes ROS accumulation [27, 28]. ROS can induce toxic stress when an imbalance occurs between ROS production and scavenging activity, even in bacteria with robust defense systems [27]. LDH release indicates cytoplasmic membrane damage and cell toxicity [29], while MDA serves as a marker of lipid peroxidation and oxidative stress [30].

Our findings suggest that the PS microplastics caused membrane damage, and this led to oxidative stress in both species due to the disruption of membrane-associated enzymes [31]. However, the specific manifestations of this membrane disruption, as indicated by measurements of ROS, LDH, and MDA, differed remarkably in these two species. *Acinetobacter* sp. showed a stronger ROS response, whereas *E. coli* exhibited higher LDH release. Both species experienced lipid peroxidation, though the effect was more pronounced in *Acinetobacter* sp. These observations suggest that PS microplastics species-specific responses to PS microplastics and the precise mechanisms underlying these differences need further investigation.

### Effect of Different Concentrations of PS Microplastics on Biofilm Formation

**Fig. 5:**
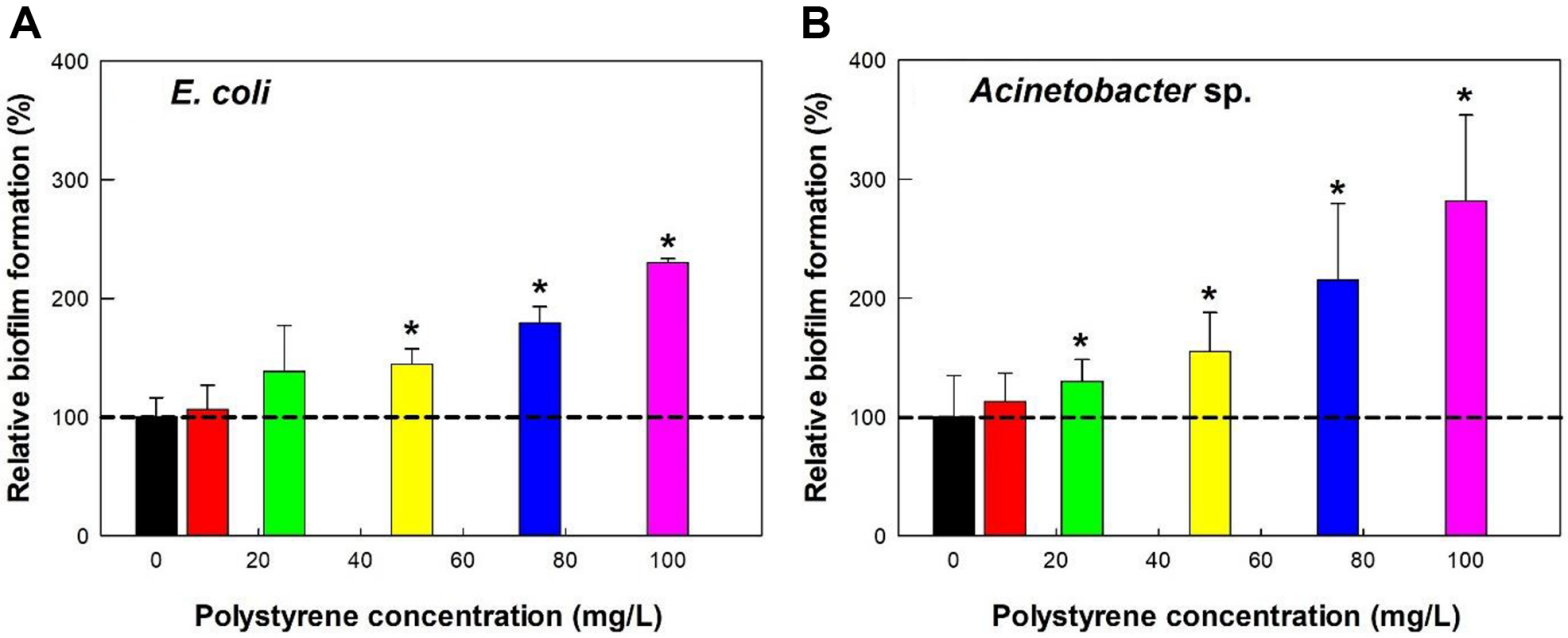
Effect of PS microplastic concentration on biofilm formation in (A) *E. coli* and (B) *Acinetobacter* sp.

Exposure to PS microplastics significantly increased biofilm formation in both *E. coli* and *Acinetobacter* sp. in a dose-dependent manner, with *Acinetobacter* sp. showing a stronger response (Fig. 5). Specifically, at a concentration of 75 mg/l PS microplastics, biofilm formation was greater in *Acinetobacter* sp. cells compared to *E. coli* cells (215 ± 64% vs. 179 ± 14%), and this trend persisted at 100 mg/l PS microplastics (282 ± 72% vs. 230 ± 3%).

The hydrodynamic diameters of *E. coli* and *Acinetobacter* sp. cells also increased in the presence of PS microplastics (Table 1). Samples of *E. coli* and *Acinetobacter* sp. cells alone had hydrodynamic diameters of 1,250 ± 128 nm and 1,800 ± 216 nm, respectively; exposure to 50 mg/l PS microplastics increased these diameters to 1,935± 374 nm and 4,819 ± 1978 nm, respectively. The larger hydrodynamic diameter of both species in the presence of PS microplastics may be related to the PS microplastic-induced formation of biofilm. In addition, the greater effect of PS microplastics on the hydrodynamic diameter of *Acinetobacter* sp. than *E. coli* may be related to the formation of larger biofilms of *Acinetobacter* sp. than *E. coli* in response to the same concentration of PS microplastics.

The enhanced biofilm formation in *Acinetobacter* sp. can be attributed to its higher production of extracellular polymeric substances (EPS) and its hydrophobic surface properties [32]. These traits, along with its strong adhesion capacity, contribute to its colonization on plastics [22, 32, 33]. The observed differences in biofilm formation between *Acinetobacter* sp. and *E. coli* on PS microplastics highlight their distinct physiological and surface interaction characteristics.

PS microplastics can promote biofilm formation by physically attaching to bacterial cells [34]. Sooriyakumar *et al*. (2022) reported that interactions between bacteria and microplastics enhanced biofilm formation [34], while Bolan *et al*. (2020) found that biofilms develop on solid microplastic surfaces through physical attachment and chemical adsorption [35]. Biofilm formation is often associated with metabolic changes and serves as an adaptive defense mechanism under stress conditions, such as limited nutrients, high temperature, and toxic agents [36]. In this study, the observed biofilm response likely reflects bacterial adaptation to the oxidative and physical stresses induced by PS microplastics. These metabolic changes may further exacerbate ROS production, as biofilm establishment often requires shifts in cellular metabolism.

### Correlations of Cell Viability, Biofilm Formation, ROS, LDH, and MDA

**Fig. 6:**
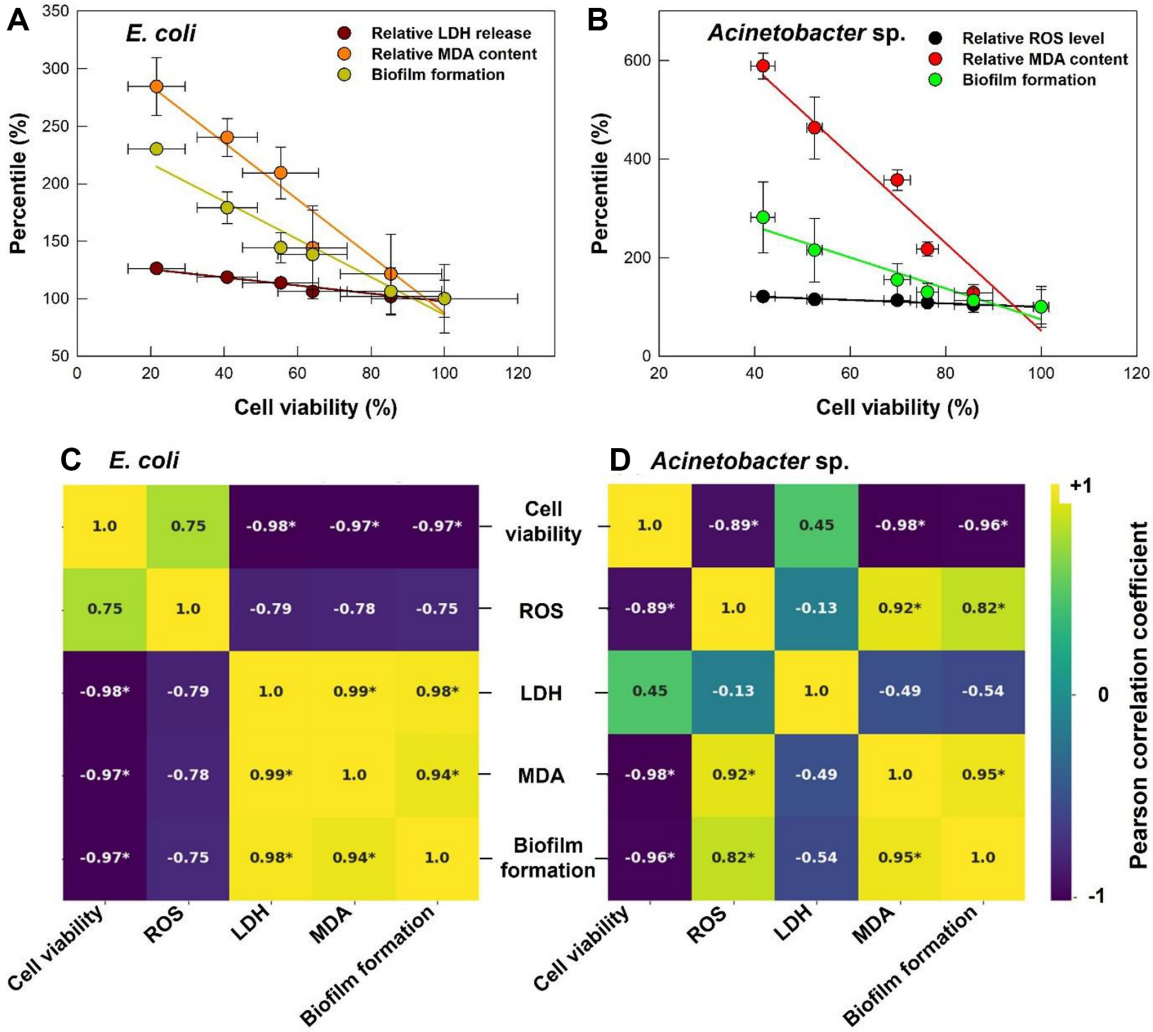
Correlation of cell viability with LDH, MDA, and biofilm formation in (A) *E. coli* and (B) with ROS, MDA, and biofilm formation in (B) *Acinetobacter* sp. for PS microplastics concentrations of 0 to 100 mg/l. Correlation heatmaps showing pairwise correlations for five variables (cell viability, ROS, LDH, MDA, and biofilm formation) in (**C**) *E. coli* and (**D**) *Acinetobacter* sp.

We performed Pearson correlation analysis to examine relationships among five markers of bacterial responses to PS microplastics in each species: cell viability, ROS, LDH, MDA, biofilm formation. In *E. coli*, cell viability exhibited strong negative correlations with LDH, MDA, and biofilm formation (all *p* < 0.05, Fig. 6A and 6C), while LDH release was positively correlated with MDA content and biofilm formation (all *p* < 0.05, Fig. 6C). On the other hand, in *Acinetobacter* sp., cell viability showed strong negative correlations with ROS, MDA, and biofilm formation (all *p* < 0.05, Fig. 6B and 6D). Notably, MDA content was strongly negatively correlated with cell viability in both species, suggesting that lipid peroxidation may be a key factor contributing to bacterial growth inhibition (Fig. 6C and 6D).

These findings revealed distinct, species-specific responses to PS microplastic exposure. For *E. coli*, the strong negative correlation between cell viability and LDH release highlights membrane integrity as a critical factor for survival. In contrast, *Acinetobacter* sp. exhibited a stronger correlation between cell viability and ROS levels, indicating that oxidative stress is more central to its response. Interestingly, the positive correlation between MDA levels and biofilm formation in both species suggests that lipid peroxidation may act as a signal for biofilm development, potentially serving as a protective mechanism against oxidative damage [37, 38]. These interactions indicate the complexity of bacterial responses to PS microplastics and provide insight into the ecological implications of microplastic pollution.

Previous studies have similarly examined the effects of microplastics on bacterial oxidative stress and viability [24, 25, 39, 40]. Ustabasi and Baysal (2020) found that exposure to PE microplastics led to growth inhibition and decreased lipid peroxidase (LPO) activity in four different bacterial strains, specifically *Staphylococcus aureus*, *Bacillus subtilis*, *E. coli*, and *Pseudomonas aeruginosa* [24]. They proposed that disrupted protein metabolism and decreased LPO activity were related to growth inhibition. Wang *et al*. (2022) observed increased LDH release from anaerobic granular sludge upon addition of 1 μm PS microplastics (75 mg/l), and that more cells appeared to be dead when exposed to these PS microplastics [41]. A recent study found that *E. coli* responded to PS microplastics with elevated levels of ROS, LDH, and MDA, and that this was accompanied by decreased cell viability [25]. Collectively, these findings support the conclusion that oxidative damage is a key mechanism driving the inhibitory effects of microplastics on bacterial growth and viability.

## Conclusion

In this study, we investigated the effects of 1,040 nm PS microplastics on *E. coli* and *Acinetobacter* sp. under a laboratory-controlled conditions. These micron-sized PS microplastics significantly inhibited growth and viability in a dose-dependent manner in both species. Although the mechanisms underlying these inhibitory effects appear to differ in *E. coli* and *Acinetobacter* sp., it is likely that oxidative stress and cell membrane damage due to the physical contact of cells with the PS microplastics may be a key factor.

Our findings demonstrate that *E. coli* and *Acinetobacter* sp. exhibit distinct responses to PS microplastic exposure, with varying degrees of oxidative stress and biofilm formation. This study provides critical insights into the toxicological effects of 1,040 nm PS microplastics on bacterial species, emphasizing their role in inducing oxidative stress and membrane disruption.

However, the laboratory experiments were performed under controlled laboratory conditions, which may not fully reflect the complexities of natural environments. Additionally, this study primarily focused on oxidative stress and biofilm-related factors, leaving other cellular pathways, such as cell death mechanisms and stress signaling, unexplored. Future studies incorporating gene and protein expression analyses are necessary to provide a more comprehensive understanding of the cellular and ecological impacts of PS microplastics.

## Figures and Tables

**Table 1:** Hydrodynamic diameters and zeta potentials of PS microplastics alone, *E. coli* cells alone, *Acinetobacter* sp. cells alone, and of *E. coli* and *Acinetobacter* sp. cells in the presence of PS microplastics.

Sample	Hydrodynamic diameter (nm)	Zeta potential (mV)
1,040 nm PS microplastics	1,100 ± 93	–61.3 ± 0.5
*E. coli* alone	1,250 ± 128	–26.7 ± 0.7
*Acinetobacter* sp. alone	1,800 ± 216	–14.0 ± 0.2
*E. coli* with PS microplastics	1,935 ± 374	–21.4 ± 0.2
*Acinetobacter* sp. with PS microplastics	4,819 ± 1,978	–15.1 ± 0.7
